# Field evaluation of a novaluron-based larvicide tablet (MOSQINOk^®^ 0.8P) in irrigated rice agroecosystems of The Gambia: a controlled quasi-experimental study

**DOI:** 10.1186/s13071-026-07501-2

**Published:** 2026-07-01

**Authors:** Ebrima A. Jawara, Momodou T. Nyassi, Sainey Sanneh, Wandifa Samateh, Balla Gibba, Samuel S. Gomez, Harouna M. Soumare, Abu Imroz Ali, Shakir Al-Zaidi

**Affiliations:** 1 RUSSELL IPM Ltd, Flintshire,, Deeside, CH5 2NU UK; 2 Directorate of Health Services, Ministry of Health, Banjul, The Gambia; 3 Directorate of Health Research, Ministry of Health, Banjul, The Gambia; 4 National Malaria Control Programme (NMCP), Ministry of Health, Kanifing, The Gambia; 5 Medical Research Council Unit The Gambia at LSHTM, Serekunda, The Gambia

**Keywords:** Larval source management, Insect growth regulator, Novaluron, Irrigated rice, *Anopheles*, Malaria, The Gambia

## Abstract

**Background:**

Larval source management remains a potentially important supplementary strategy for malaria vector control, particularly in irrigated agroecosystems, where mosquito breeding habitats are relatively stable and predictable. This study evaluated the operational effectiveness of a novaluron-based larvicide tablet (MOSQINOk^®^ 0.8P) for reducing mosquito larval and adult densities in irrigated rice-growing communities in The Gambia.

**Methods:**

A quasi-experimental controlled field study was conducted between September and December 2025 in two irrigated rice-growing communities in the Central River Region of The Gambia. Janjangbureh was assigned as the intervention site and Sankuli Kunda as the untreated control. Larval habitats were monitored weekly, while adult mosquitoes were collected biweekly using CDC light traps. Difference-in-differences analyses using negative binomial generalized estimating equation models were used to estimate intervention effects.

**Results:**

MOSQINOk^®^ application was associated with a significant reduction in total larval density per dip (IRR = 0.459, 95% CI 0.216–0.974; *p* = 0.042), corresponding to an estimated 54.1% reduction relative to the control site. Late instar larval densities also declined in the intervention arm (IRR = 0.485, 95% CI 0.186–1.260), although this was not statistically significant. Pupae were nearly absent in treated habitats following larvicide application. Adult *Anopheles* densities also declined in the intervention arm, with difference-in-differences models showing reductions in overall adult *Anopheles* (IRR = 0.385, 95% CI 0.121–1.225), *Anopheles gambiae* s.l. (IRR = 0.316, 95% CI 0.098–1.014), and *Anopheles funestus* (IRR = 0.443, 95% CI 0.107–1.834).

**Conclusions:**

These findings provide preliminary evidence that MOSQINOk^®^ 0.8P can contribute to suppression of mosquito larval populations and may reduce adult vector densities in irrigated rice agroecosystems. Larger and longer-term studies are warranted to confirm effectiveness and scalability.

**Graphical Abstract:**

**Figure:**
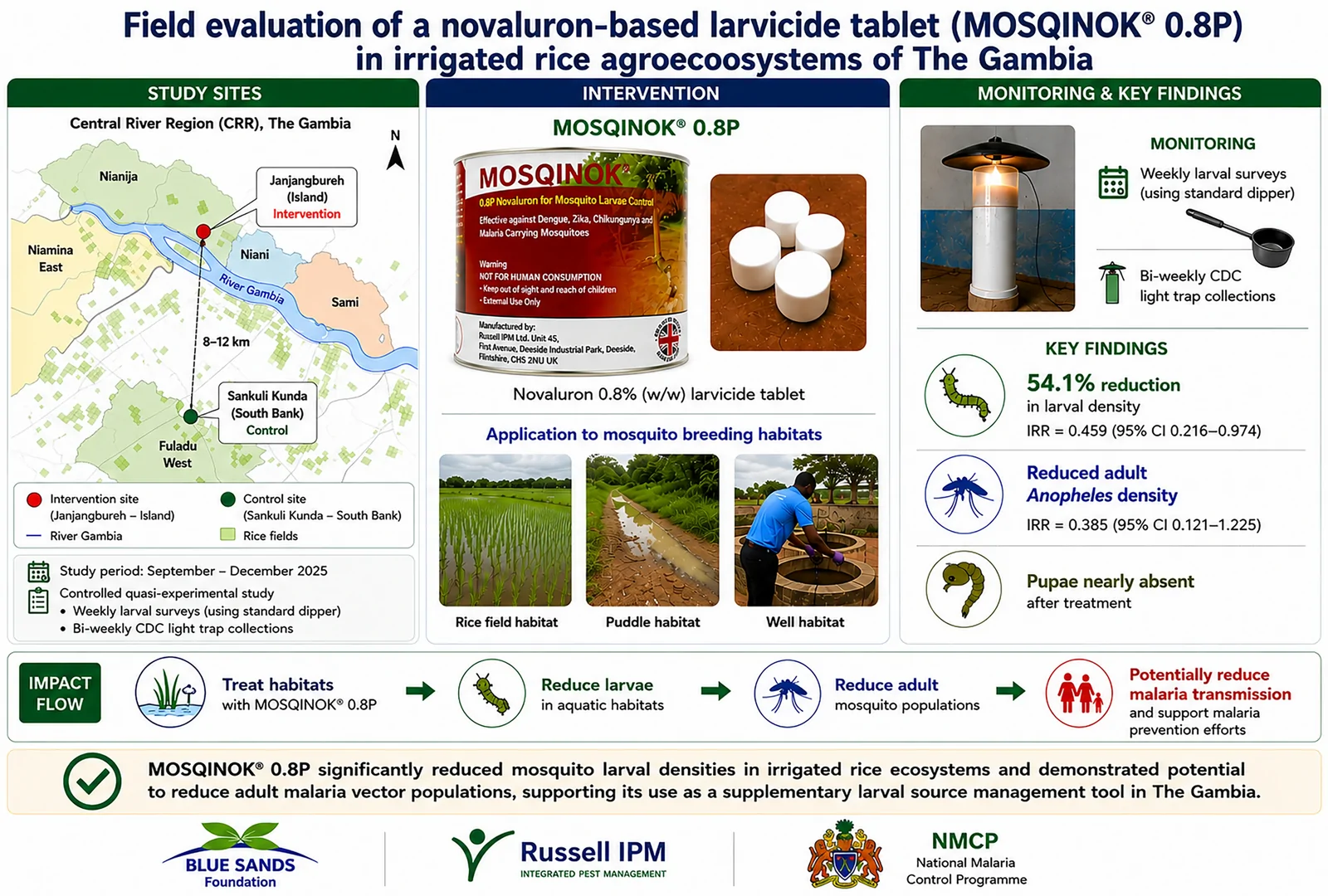

**Supplementary Information:**

The online version contains supplementary material available at https://doi.org/10.1186/s13071-026-07501-2.

## Background

Despite substantial progress in malaria control, residual malaria transmission remains a major challenge in many endemic settings, particularly where mosquitoes exploit breeding habitats that are not adequately targeted by conventional interventions, such as long-lasting insecticidal nets (LLINs) and indoor residual spraying (IRS) [1–4]. In irrigated rice agroecosystems, persistent aquatic habitats can support continuous mosquito breeding and contribute to sustained malaria transmission [5–7].

Larval source management (LSM) is recommended by the World Health Organization as a supplementary vector control strategy in settings, where breeding habitats are relatively fixed, findable, and manageable [8]. Interest in LSM has increased in response to operational challenges affecting conventional interventions and increasing insecticide resistance among malaria vectors [9]. Rice cultivation environments may represent particularly suitable settings for LSM, because breeding sites are often predictable and accessible for repeated intervention [6, 8].

Novaluron is an insect growth regulator that acts by inhibiting chitin synthesis, disrupting larval development and preventing successful moulting and pupation [10–12]. Previous studies have demonstrated promising effects of insect growth regulators against mosquito larvae; however, evidence from operational field evaluations in irrigated rice ecosystems of West Africa remains limited [10–12]. In The Gambia, persistent vector breeding habitats associated with irrigated agriculture may continue to contribute to malaria transmission despite reductions in malaria burden [13–15].

This study evaluated the effectiveness of a novaluron-based larvicide tablet (MOSQINOk^®^ 0.8P) for reducing mosquito larval and adult densities in irrigated rice-growing communities in the Central River Region of The Gambia.

**Fig. 1:**
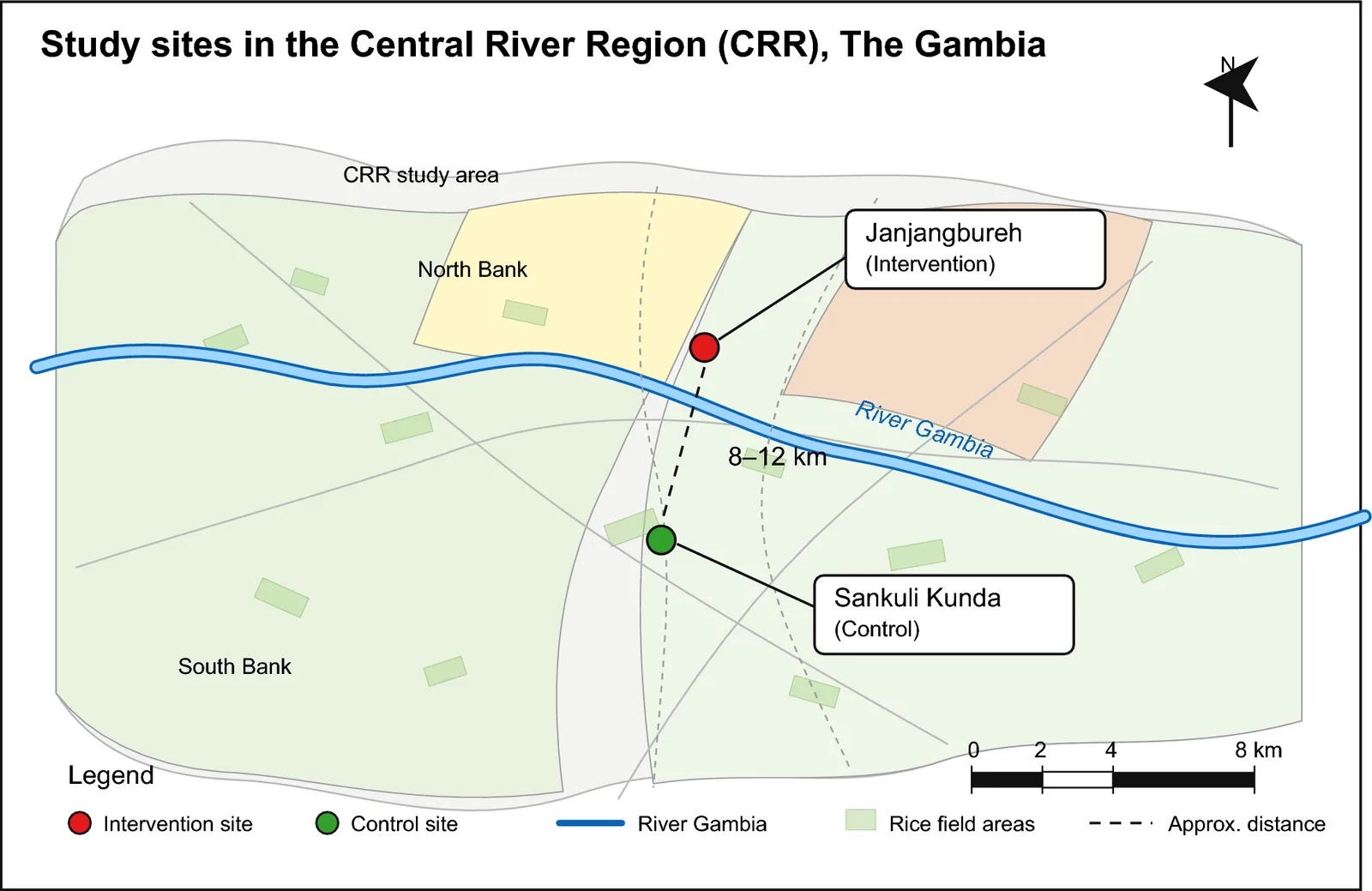
Location of the intervention site (Janjangbureh Island) and control site (Sankuli Kunda, south bank) in the Central River Region (CRR), The Gambia. The River Gambia separates the two study villages, which are approximately 8–12 km apart and hydrologically independent

This study was conducted in two villages in the Central River Region (CRR) of The Gambia: Janjangbureh (intervention) and Sankuli Kunda (control). The communities were located approximately 8–12 km apart and had no direct hydrological connection. Both communities are characterized by irrigated rice cultivation systems and comparable rural settlement patterns, supporting their suitability for controlled comparison (Fig. 1).

A controlled quasi-experimental difference-in-differences (DiD) design was used, consisting of a 1-week baseline period followed by 12 weeks of post-intervention monitoring.

MOSQINOk^®^ 0.8P tablets were applied directly to aquatic habitats in the intervention arm at a standardised operational dose of one tablet per approximately 200 L of estimated water volume, estimated from habitat surface area and mean depth measurements. The tablet formulation is designed to gradually release novaluron into relatively stagnant aquatic habitats, providing sustained exposure of immature mosquito stages to the active ingredient. Fixed larval monitoring sites were sampled weekly using standard dipping methods, while adult mosquitoes were collected biweekly using CDC light traps at sentinel households.

The primary outcome was total larval density per dip. Secondary outcomes included late instar (L3–L4) density per dip and adult mosquito densities per trap-night.

Difference-in-differences analyses were conducted using negative binomial generalized estimating equation (GEE) models with a log link to account for overdispersed count data and repeated observations (Table 1). Sampling effort was incorporated using log-transformed offsets based on the number of dips per site visit for larval outcomes and trap-nights for adult collections. Effect estimates are presented as incidence rate ratios (IRR) with 95% confidence intervals. Analyses were conducted in R version 4.3.2 using the geepack package.

**Table 1:** Baseline characteristics and entomological indicators by study arm

Characteristic	Control	Intervention
Sampling sites (*n*)	20	20
Total dips at baseline	390	400
Mean larval density per dip (SD)	1.94 (2.40)	2.66 (1.82)
Trap locations (*n*)	5	5
Trap-nights at baseline	15	15
Mean adult *Anopheles* per trap-night (SD)	3.40 (3.66)	2.80 (2.43)

A total of 40 fixed larval sampling sites (20 per arm) were monitored throughout the study period. Baseline mean larval density per dip was 2.66 in the intervention arm and 1.94 in the control arm (Table 2). Adult mosquito surveillance was conducted at five sentinel households per arm using CDC light traps.

**Table 2:** Difference-in-differences estimates for larval and adult mosquito outcomes following MOSQINOk^®^ 0.8P application

Outcome	IRR	95% CI	*p* value	Relative reduction (%)
Total larvae per dip	0.459	0.216–0.974	0.042	54.1
Late instars (L3–L4) per dip	0.485	0.186–1.260	0.137	51.5
Adult *Anopheles* per trap-night	0.385	0.121–1.225	0.106	61.5
Adult *An. gambiae* s.l. per trap-night	0.316	0.098–1.014	0.053	68.4
Adult *An. funestus* per trap-night	0.443	0.107–1.834	0.263	55.7

**Fig. 2:**
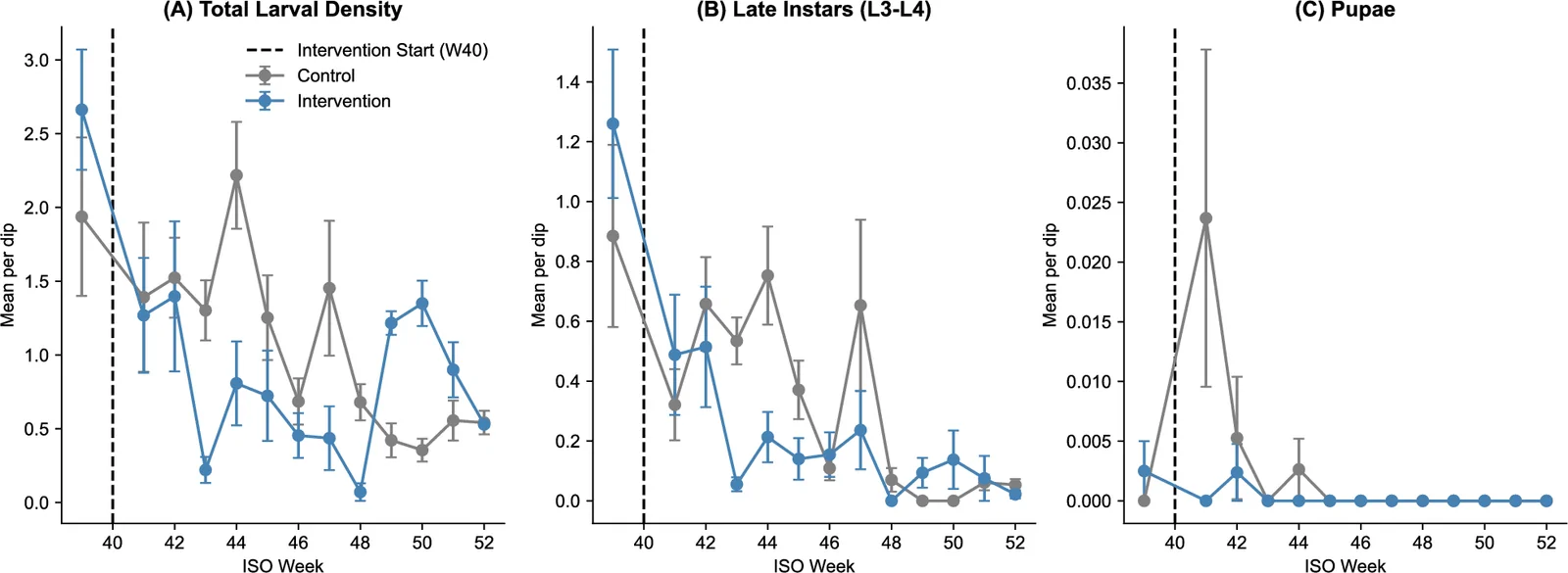
Weekly larval dynamics by study arm: **A** total larval density per dip, **B** late instar (L3–L4) density per dip, and **C** pupal density per dip. Values represent site-week means with site-clustered 95% confidence intervals. The dashed vertical line indicates the timing of larvicide application at ISO week 40

Following application of MOSQINOk^®^ 0.8P, larval density declined relative to the control arm, with sustained reductions observed in treated sites during the post-intervention period (Fig. 2A). The difference-in-differences model showed a significant reduction in total larval density (IRR 0.459; 95% CI 0.216–0.974; *p* = 0.042), corresponding to a 54.1% reduction relative to control trends.

Late instar (L3–L4) density also declined in the intervention arm following treatment (Fig. 2B), although estimates were imprecise (IRR 0.485; 95% CI 0.186–1.260; *p* = 0.137).

Pupal density declined markedly in treated habitats (Fig. 2C). More than 90% of post-intervention site-week observations recorded zero pupae in the intervention arm, whereas pupae continued to be detected intermittently in the control arm. Across the post-intervention period, only one pupa was recorded in treated habitats compared with 12 in the control arm.

Adult mosquito densities also declined following intervention and were broadly consistent with observed larval reductions. Overall adult *Anopheles* abundance declined relative to the control arm (IRR 0.385; 95% CI 0.121–1.225; *p *= 0.106), corresponding to a 61.5% reduction. *Anopheles gambiae* s.l. abundance also declined (IRR 0.316; 95% CI 0.098–1.014; *p* = 0.053). *Anopheles funestus* densities showed a similar directional trend (IRR 0.443; 95% CI 0.107–1.834; *p* = 0.263), although confidence intervals were wide. Additional temporal trends are presented in Supplementary Figure S1 (Additional file 1).

This study provides field evidence that application of a novaluron-based larvicide formulation was associated with reduced mosquito larval densities in irrigated rice agroecosystems of The Gambia. The significant reduction in total larval density and marked suppression of pupae are biologically consistent with the expected mode of action of insect growth regulators, which inhibit chitin synthesis and interfere with normal larval development and successful emergence [10–12].

The near absence of pupae following intervention is particularly important, because insect growth regulators generally do not produce rapid knockdown of early larval stages but instead interrupt developmental progression to adulthood [10, 11]. Continued detection of some early instars during the post-intervention period may, therefore, reflect ongoing oviposition rather than treatment failure. Similar patterns have been observed in previous evaluations of insect growth regulators and larval source management interventions [10, 11]. The magnitude of larval reduction observed in the present study was also comparable to reductions reported in previous field evaluations of novaluron and other insect growth regulator formulations under operational conditions [10, 11].

Although reductions in adult mosquito abundance were associated with wide confidence intervals and limited statistical precision, the observed trends were broadly consistent with reductions in immature mosquito stages. Previous studies have similarly reported that reductions in larval habitat productivity may contribute to subsequent reductions in adult vector populations [6, 16, 17]. The reduction observed for *Anopheles gambiae* s.l. is noteworthy, because this species complex remains an important malaria vector in The Gambia and across much of sub-Saharan Africa [4, 15].

Irrigated rice ecosystems may represent suitable environments for larval source management, because aquatic habitats are relatively stable and identifiable compared with more dispersed temporary breeding sites [6–8]. WHO recommendations indicate that larval interventions may be particularly useful, where breeding habitats can be reliably located and repeatedly treated [8]. The operational use of tablet formulations may also offer practical advantages for routine implementation in agricultural settings.

This study has limitations. The quasi-experimental design involving a single intervention and control community limits causal inference and may introduce village-level confounding. The short baseline period and limited adult sampling intensity may also have reduced statistical power for adult mosquito outcomes. Furthermore, mosquito movement between surrounding areas may have influenced observed adult densities. Environmental factors such as rainfall variation and habitat turnover were not explicitly modelled and may also have influenced mosquito population dynamics. Larger multi-site evaluations with longer follow-up periods are needed to confirm effectiveness and operational scalability across different ecological settings. Overall, these findings provide preliminary evidence supporting the potential role of novaluron-based larval source management as a complementary vector control strategy in agricultural settings.

## Supplementary Information

Supplementary Material 1.

## Data Availability

Data supporting the main conclusions of this study are included in the manuscript.

## Abbreviations

CDC: Centers for disease control and prevention
CI: Confidence interval
CRR: Central river region
DiD: Difference-in-differences
GEE: Generalized estimating equations
IGR: Insect growth regulator
IRR: Incidence rate ratio
IRS: Indoor residual spraying
LLINs: Long-lasting insecticidal nets
LSM: Larval source management
MRCG: Medical Research Council Unit The Gambia at LSHTM
NMCP: National Malaria Control Programme
SD: Standard deviation
WHO: World Health Organization
