# Supplementary material for: Field evaluation of a novaluron-based larvicide tablet (MOSQINOk® 0.8P) in irrigated rice agroecosystems of The Gambia: a controlled quasi-experimental study
Source: Parasit Vectors. 2026 Jul 1;19:420. doi: 10.1186/s13071-026-07501-2 (PMC13625327; doi:10.1186/s13071-026-07501-2)
Supplement: Supplementary file 1 [file 13071_2026_7501_MOESM1_ESM.pdf]

Additional file 2: Supplementary Figures S1

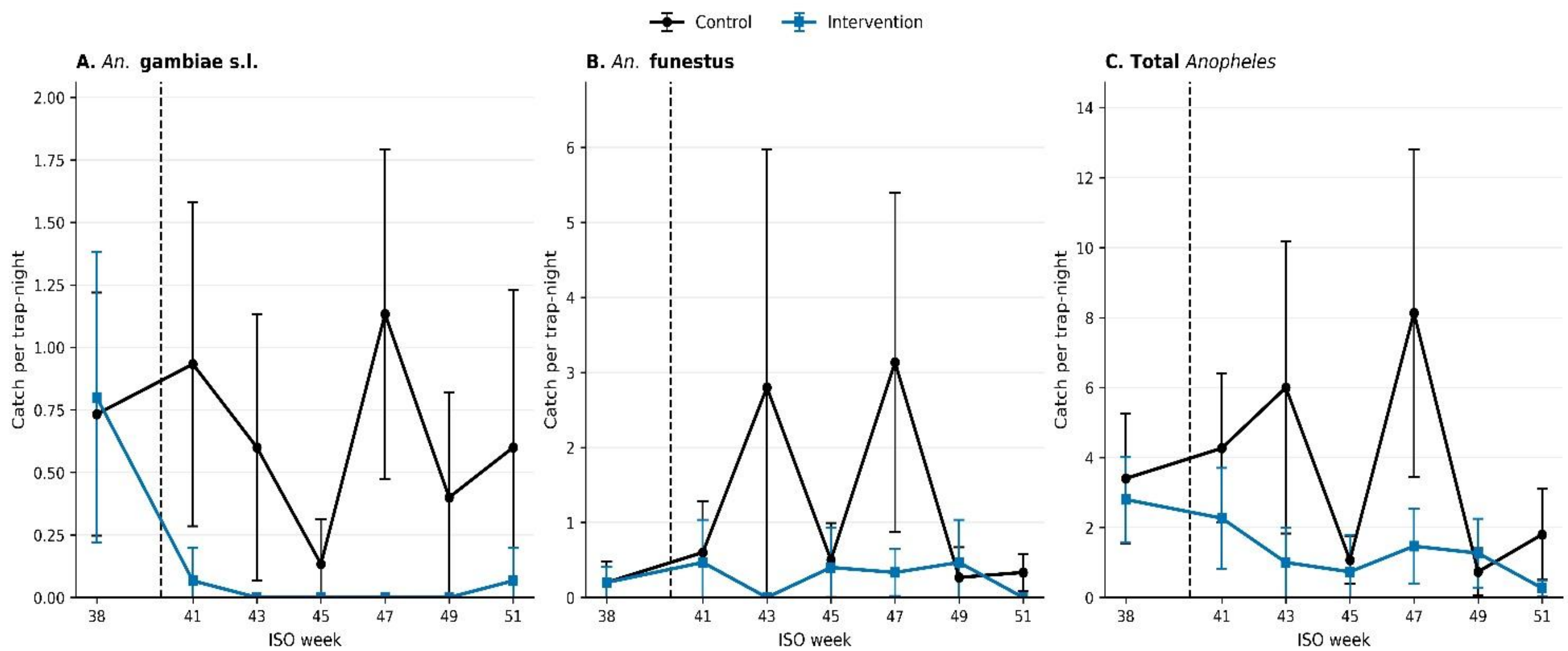

Supplementary Figure S1. Weekly trends in adult mosquito densities collected using CDC light traps in intervention and control communities during the study period. Panels show mean trap-night densities of (A) *Anopheles gambiae s.l.*, (B) *Anopheles funestus*, and (C) total *Anopheles* mosquitoes by ISO week. The vertical dashed line indicates the timing of MOSQINOk® 0.8P application (ISO week 40). Error bars represent 95% confidence intervals.
